# Exploring the Sensitivity Limits of Neuronal Current Imaging With MRI and MEG in the Human Brain

**DOI:** 10.1002/hbm.70624

**Published:** 2026-08-07

**Authors:** Milena Capiglioni, Davide Tabarelli, Stefano Tambalo, Federico Turco, Roland Wiest, Jorge Jovicich

**Affiliations:** ^1^ Support Center for Advanced Neuroimaging (SCAN), Inselspital Institute for Diagnostic and Interventional Neuroradiology Bern Bern Switzerland; ^2^ High‐Field MR Center Max Planck Institute for Biological Cybernetics Tübingen Germany; ^3^ Center for Mind and Brain Sciences (CIMeC) University of Trento Rovereto Italy; ^4^ Department of Physics University of Trento Trento Italy; ^5^ Trento Institute for Fundamental Physics and Applications (TIFPA‐INFN), University of Trento Trento Italy; ^6^ Department of Physics University of Torino Torino Italy; ^7^ Department of Molecular Biotechnology and Health Sciences University of Torino Torino Italy

**Keywords:** MEG, multimodal imaging, neuroelectrical oscillations, non‐BOLD fMRI, spin‐lock fMRI

## Abstract

Conventional BOLD‐fMRI relies on hemodynamic responses that are temporally and spatially indirect markers of neural activity. Developing alternative contrasts, sensitive to neuroelectrical phenomena, is a critical challenge in brain imaging. Spin‐lock (SL) fMRI has shown promise in phantom studies for detecting magnetic field changes associated with neuronal activity, but its in‐vivo sensitivity and practicality remain unclear. This study evaluated whether SL contrast can effectively detect and localize human neuronal activation, benchmarked against complementary functional modalities, magnetoencephalography (MEG) and 3T BOLD‐fMRI, to assess the sensitivity of MR‐based neuronal current imaging. Thirteen healthy young volunteers underwent SL‐based imaging during 8 Hz visual stimulation, along with BOLD and MEG acquisitions. Subjects viewed quadrant‐checkerboard stimuli to elicit localized cortical responses. Two balanced SL contrast mechanisms, rotary excitation (REX) and stimulus‐induced rotary saturation (SIRS), were employed. Postprocessing targeted stimulus‐locked signal fluctuations using a regression‐filtering‐rectification strategy. Phantom experiments tested sensitivity and analysis pipeline performance. MEG revealed robust stimulus‐locked responses in the occipital cortex, with estimated local magnetic field amplitudes of ~0.07 nT. Conventional BOLD‐fMRI confirmed reliable hemodynamic activation. In contrast, neither balanced REX nor balanced SIRS produced consistent stimulus‐related activation in vivo. Phantom experiments subsequently yielded detection thresholds of 0.2 nT for REX and 0.6 nT for SIRS, exceeding the MEG‐estimated physiological field amplitudes. Under the present experimental conditions, the tested spin‐lock fMRI implementations did not achieve sufficient sensitivity for reliable in vivo detection of neuronal magnetic fields at 3T. Phantom and MEG‐based estimates indicate that physiological field amplitudes in the visual cortex lie below current detection limits. These findings establish quantitative constraints on direct neuronal current imaging with MRI and provide a benchmark for future methodological developments aimed at bridging electrophysiology and functional MRI.

## Key Points

- We assessed spin‐lock fMRI sensitivity using combined SL‐fMRI, BOLD‐fMRI, MEG, and phantom measurements during visual stimulation.
- MEG and BOLD‐fMRI confirmed robust neuronal and hemodynamic activation in the visual cortex.
- SL‐fMRI did not achieve reliable in vivo detection of neuronal magnetic fields; phantom sensitivity limits exceeded MEG‐estimated physiological field amplitudes.

## Graphical abstract

Combined spin‐lock fMRI, MEG, BOLD‐fMRI, and phantom experiments reveal that physiological neuronal magnetic fields lie below the in vivo detection threshold of the current spin‐lock implementations at 3T, defining quantitative sensitivity limits for MR‐based neuronal current imaging.

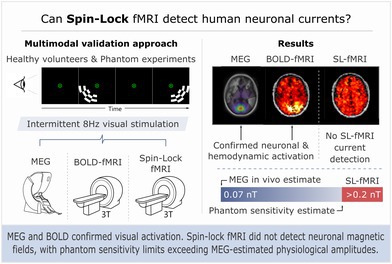

## 1 Introduction

The ability to non‐invasively image neuronal activity with high spatial and temporal resolution remains a central challenge in neuroimaging. While blood oxygen level dependent (BOLD) functional MRI has revolutionized the study of human brain function, it gives only an indirect measure of neural activity through neurovascular coupling (Logothetis 2008). In contrast, electroencephalography (EEG) and magnetoencephalography (MEG) measure neuronal currents directly via electric and magnetic fields, but are limited by poor spatial resolution and source ambiguity due to the ill‐posed inverse problem (Ahlfors et al. 2010; Cicmil et al. 2014; Gross et al. 2013; Hämäläinen et al. 1993; Michel and Murray 2012).

To address this gap, researchers have explored non‐BOLD MRI methods aimed at directly detecting neuronal magnetic fields. Early efforts focused on capturing small neuronal field‐induced phase shifts in gradient‐echo signals (Bodurka and Bandettini 2002; Petridou et al. 2006), though in vivo validation proved difficult (Chu et al. 2004; Parkes et al. 2007). More recently, a 2D line‐scanning method claimed high‐temporal‐resolution detection of neuronal currents, but initial reports were later retracted, and findings could not be reproduced (Choi et al. 2024; Phi Van et al. 2024).

Another emerging alternative is spin‐lock functional MRI (SL‐fMRI). First proposed in 2008 (Witzel et al. 2008), SL‐fMRI exploits the resonance interaction between spin‐locked magnetization and oscillating neuronal magnetic fields to produce functional contrast. Because the signal depends on the initial phase of the neuronal field at the onset of the spin‐lock pulse (Capiglioni et al. 2022), on‐resonance SL‐fMRI acquisitions are expected to exhibit increased signal variance compared with off‐resonance acquisitions, allowing separation from conventional BOLD effects. Phantom studies have demonstrated that SL‐fMRI can detect neuron‐like magnetic field fluctuations with sub‐nanotesla sensitivities (Halpern‐Manners et al. 2010; Sveinsson et al. 2020). However, in vivo application of SL‐fMRI remains limited. Promising results have been reported in epilepsy, where neuronal activity is expected to be stronger (Kiefer et al. 2016; Unger et al. 2021), while studies in healthy volunteers have shown only group‐level evidence during visual stimulation (Capiglioni et al. 2025; Truong et al. 2019), with limited or absent detection at the individual level (Capiglioni et al. 2025). These findings highlight persistent questions regarding in vivo sensitivity, robustness to field imperfections, and the ability of SL‐fMRI to resolve physiologically relevant neuronal signals.

Two implementations of SL‐fMRI have emerged: stimulus‐induced rotary saturation (SIRS) (Witzel et al. 2008) and rotary excitation (REX) (Truong et al. 2019). In SIRS, magnetization is tipped into the transverse plane, spin‐locked, and then tipped back up, such that neuronal field interactions during the spin‐lock period are encoded as a saturation of longitudinal magnetization. In contrast, REX omits the tip‐up pulse and instead detects transverse magnetization generated during the spin‐lock period, followed by a slice‐selective excitation applied after the spin‐lock block. For the small effective flip angles expected from neuronal magnetic fields, REX is expected to provide higher sensitivity than SIRS. Composite and balanced SL preparations, adapted from T1ρ mapping protocols (Wáng et al. 2015; Witschey et al. 2007) have been introduced to mitigate sensitivity to B0 and B1 inhomogeneities. These approaches exploit the symmetric effect of RF imperfections on the preparation pulses, thereby improving baseline stability in the absence of neuronal field effects (Capiglioni et al. 2022; Gram et al. 2022). Since REX does not include a tip‐up pulse, it cannot fully exploit this symmetry‐based compensation strategy, thus adiabatic tip‐down pulses are commonly used to improve robustness to field imperfections (Gram et al. 2022). However, the relative performance of these balanced SIRS and REX implementations for detecting in vivo neuronal signals has yet to be established.

In this study, we evaluate and compare the functional sensitivity of both balanced SIRS and REX SL‐fMRI sequences during visual stimulation in healthy volunteers using a 3T clinical scanner. We used BOLD‐fMRI and MEG as benchmark modalities, while phantom experiments with identical acquisition protocols provide in vitro sensitivity estimates, helping to contextualize in vivo findings.

## 2 Materials and Methods

The study was designed as a three‐stage multimodal validation framework. First, in vivo SL‐fMRI, BOLD‐fMRI, and MEG data were acquired during visual stimulation to assess functional sensitivity under realistic experimental conditions. Second, MEG recordings were used to estimate stimulus‐locked neuronal magnetic field amplitudes in the visual cortex. Third, dedicated phantom experiments employing identical acquisition and analysis pipelines were conducted to quantify the minimum detectable field amplitudes for each spin‐lock implementation. This integrated approach enabled direct comparison between physiological signal magnitudes and sequence‐specific sensitivity limits. Visual stimulation was selected because it elicits robust and spatially well‐characterized responses in the primary visual cortex, making it suitable for MEG source reconstruction (Cicmil et al. 2014) and cross‐modal comparison with MRI. In addition, periodic visual stimulation produces a controlled frequency response, which is required for the spin‐lock method to target frequency‐specific neuronal activity (Witzel et al. 2008). This paradigm also enables frequency tagging, a well‐established M/EEG approach that produces responses at a defined stimulation frequency (Retter and Schiltz 2025).

In this study, the visual stimulus flickered at 8 Hz, while the spin‐lock frequency was set at 16 Hz to target the first harmonic of the evoked response, which showed a prominent response in MEG measurements (Retter and Schiltz 2025).

### 2.1 Human Experiments

Thirteen healthy participants (mean age 26.1 ± 5.9, range 20–43 years) underwent scanning on a 3T MRI scanner and magnetoencephalography. The study was approved by the Ethical Committee of the University of Trento, and all volunteers provided written informed consent prior to participation in the study. All participants had normal, or corrected to normal, visual acuity. The visual stimulation protocol was programmed in Psychtoolbox‐3 (http://psychtoolbox.org/) and eye‐tracker measurements were acquired in both modalities during the experiment. The MEG was performed at least 3 days before MRI to avoid the influence of residual magnetization (Gross et al. 2013). For both modalities, the participants viewed a checkerboard quadrant stimulus flickering at 8 Hz, corresponding to light and dark phases of 62.5 ms each (total period 125 ms). Stimulation blocks alternated with rest periods consisting of a black screen. The next subsections describe the details of the experiments and analysis for both modalities.

### 2.2 MEG Data Acquisition

**FIGURE 1:**
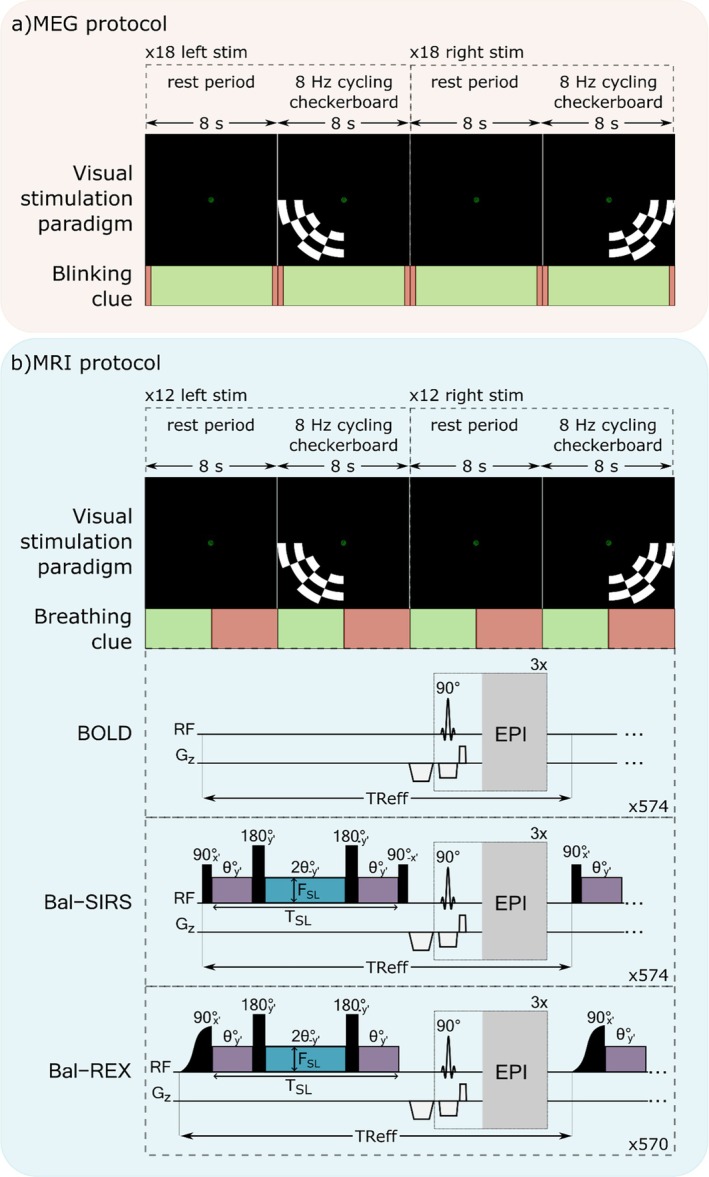
MEG and fMRI acquisition and visual stimulation protocols. (a) Experimental paradigm for MEG. 8‐s blocks of 8 Hz checkerboard quadrant stimuli and 2‐s blinking period indicated by fixation point and (b) experimental paradigm for functional MRI (three axial AC–PC slices covering the primary visual cortex). 8‐s blocks of 8 Hz checkerboard stimuli. Breathing periods of 4 s indicated by color change of the fixation point. MRI acquisition includes BOLD (equal timing to SIRS, omits SL preparation), balanced SIRS, and balanced REX. θ represents the induced rotation angle in each SL segment, and RF pulse phase polarity is indicated by color. A crusher gradient along *z* is applied after SL, followed by three consecutive EPI readouts.

We recorded MEG data in a magnetically shielded room using a 306‐channel Neuromag VectorView system (204 planar gradiometers, 102 magnetometers; MEGIN, Helsinki, Finland). Signals were sampled at 1 kHz (low pass filter at 0.3 Hz and high pass filter at 330 Hz). Participants viewed flickering checkerboards (8 Hz) while maintaining central fixation during alternating 8 s stimulation and 8 s rest periods. The first 18 flickering periods occurred in the lower left and the second 18 in the lower right visual field (Figure 1a). At each transition between stimulation conditions, a 2 s interval allowed participants to blink. For each subject, we repeated the full experiment twice. During data acquisition, we continuously monitored head position using five head position indicator (HPI) coils. Before preprocessing, we realigned the head position to a reference common position using the signal source separation method (SSS) (Taulu and Kajola 2005), minimizing potential errors due to head movements. We recorded eye movements monocularly at 1 kHz using an MEG compatible eye tracking system to subsequently reject eye‐movement related artifacts.

### 2.3 MEG Preprocessing and Analysis

We preprocessed and analyzed MEG data using MNE‐Python (Gramfort et al. 2014). This involved identification and removal of noisy channels and artifact‐contaminated epochs, rejecting and correcting residual ocular and muscle artifacts with independent component analysis, and computing power spectra using a discrete prolate spherical sequences‐based multitaper method (4–42 Hz; epochs length: 4 s; bandwidth: 2 Hz). Power spectra were computed and analyzed for each subject at the sensor level as overall power spectral density and a time frequency map, with a 20 ms resolution.

We estimated the source of activation in the brain using a dynamic imaging of coherent sources approach (Gross et al. 2001). The Fourier parameters were the same, leading to a spectral resolution of 0.25 Hz. We based cortical reconstruction on individual anatomical models derived from each participant's T1‐weighted MRI and FreeSurfer surfaces. We then computed the ratio of activity between stimulation and rest, and tested it statistically using a non‐parametric permutation statistics approach to provide significant activation maps at the source level. The group source level activation maps were obtained from 11 out of 13 participants. One subject was excluded due to unsuccessful anatomical reconstruction from the T1‐weighted scan, and another due to incorrect stimulation timing. The latter subject has been excluded also from the power spectra analysis.

Finally, we estimated local magnetic field amplitudes in the occipital cortex from MEG recordings following the approximate approach reported by Bodurka and Bandettini (2002). In this framework, scalp‐recorded signals are approximated as arising from equivalent current dipoles within a 1 mm^2^ voxel, containing on the order of 5 × 10^5^ aligned pyramidal neurons oriented tangentially to the scalp, as commonly assumed in MEG/EEG source modeling (Hämäläinen et al. 1993). Given source–sensor distances of approximately 2–4 cm and source–voxel distances of approximately 1–2 mm, a distance‐based scaling derived from the Biot–Savart law yields an approximate factor of 10^3^ between cortical and sensor‐level magnetic field strengths (Bodurka and Bandettini 2002). Based on this, each participant's MEG data was band‐pass filtered around the frequency of interest 𝑓 using a narrow bandwidth (Δ*f* = 1 Hz). We then computed the Hilbert envelope at the selected frequency and estimated the local magnetic field amplitude by multiplying the average Hilbert envelope by a factor of 10^3^. This provides an approximate conversion from sensor‐level measurements to cortical field strength. We applied this procedure separately for left‐ and right‐field stimulation conditions and subsequently averaged across subjects to obtain group‐level estimates. The resulting values should be interpreted as conservative, order‐of‐magnitude estimates of cortical field strength, as they assume purely tangential currents and do not account for phase cancelation, nonlinearities, or other effects that would further reduce the measured sensor‐level amplitudes.

Further details regarding acquisition, preprocessing, and modeling procedures appear in Supporting Information 1.

### 2.4 MRI Data Acquisitions

#### 2.4.1 In Vivo MRI Experiments

We acquired MRI data on a 3T clinical scanner (MAGNETOM Prisma, Siemens Healthineers AG, Erlangen, Germany) equipped with a 64 channel head–neck receive coil. After standard T1‐weighted MPRAGE images for anatomical reference, functional MRI data was acquired while participants performed a passive visual task designed to match the MEG experiment, with timing adjusted to fit MRI acquisition constraints. The visual stimuli were identical to those used in MEG: a flickering black‐and‐white checkerboard presented in either the lower left or right visual field. For MRI, each checkerboard was displayed for 8 s at 8 Hz, alternating with 8‐s blank screens. Initial MEG measurements showed a prominent response at the second harmonic (16 Hz) of the visual stimulation, motivating the use of a 16 Hz spin‐lock frequency in the MRI experiments. The first 12 stimulation blocks targeted the lower left visual field, followed by 12 blocks in the lower right. A central fixation cross was displayed throughout the experiment, changing color every 4 s to cue breathing (Figure 1b). The controlled breathing was to ensure that the breathing frequency didn't match the stimulation frequency (1/16 = 0.0625 Hz), and that it could therefore be eliminated by high‐pass filtering.

This paradigm was repeated for three functional MR sequences: balanced SIRS (Bal‐SIRS) (Capiglioni et al. 2022; Witzel et al. 2008), balanced REX (Bal‐REX) (Gram et al. 2022; Truong et al. 2019), and conventional BOLD‐fMRI (Figure 1b). All sequences used a shared EPI readout covering the primary visual cortex with three AC–PC oriented axial slices (matrix size = 64 × 64 × 3; voxel size = 3.4 × 3.4 × 5 mm^3^; flip angle = 90°; TE = 29.8 ms; bandwidth = 1950 Hz/pixel, slice gap = 5 mm). For SIRS and REX, the SL frequency was set at 16 Hz for on‐resonance condition and at 36 Hz to have an off‐resonance control condition expected to give no functional sensitivity. Bloch simulations of the SL effect (Capiglioni et al. 2022) were used to determine the optimal spin‐lock duration (TSL), which was fixed at 112 ms for all acquisitions and frequencies, as this value maximized signal variation while maintaining consistent T₁ρ weighting across conditions (see Supporting Information 2). The BOLD fMRI sequence used the same repetition time (TR) of 334 ms as the SL acquisitions, leaving the spin‐lock preparation period empty to ensure comparable timing and signal evolution between sequences. The relatively short repetition time (TR = 334 ms) was selected to maximize the number of repetitions within a fixed scan duration and spatial coverage from three slices (5 mm each), which were deemed sufficient to cover the human primary visual cortex. Each acquisition began after 12 dummy scans, yielding a total duration of 6.4 min per sequence.

#### 2.4.2 Phantom MRI Experiments

**FIGURE 2:**
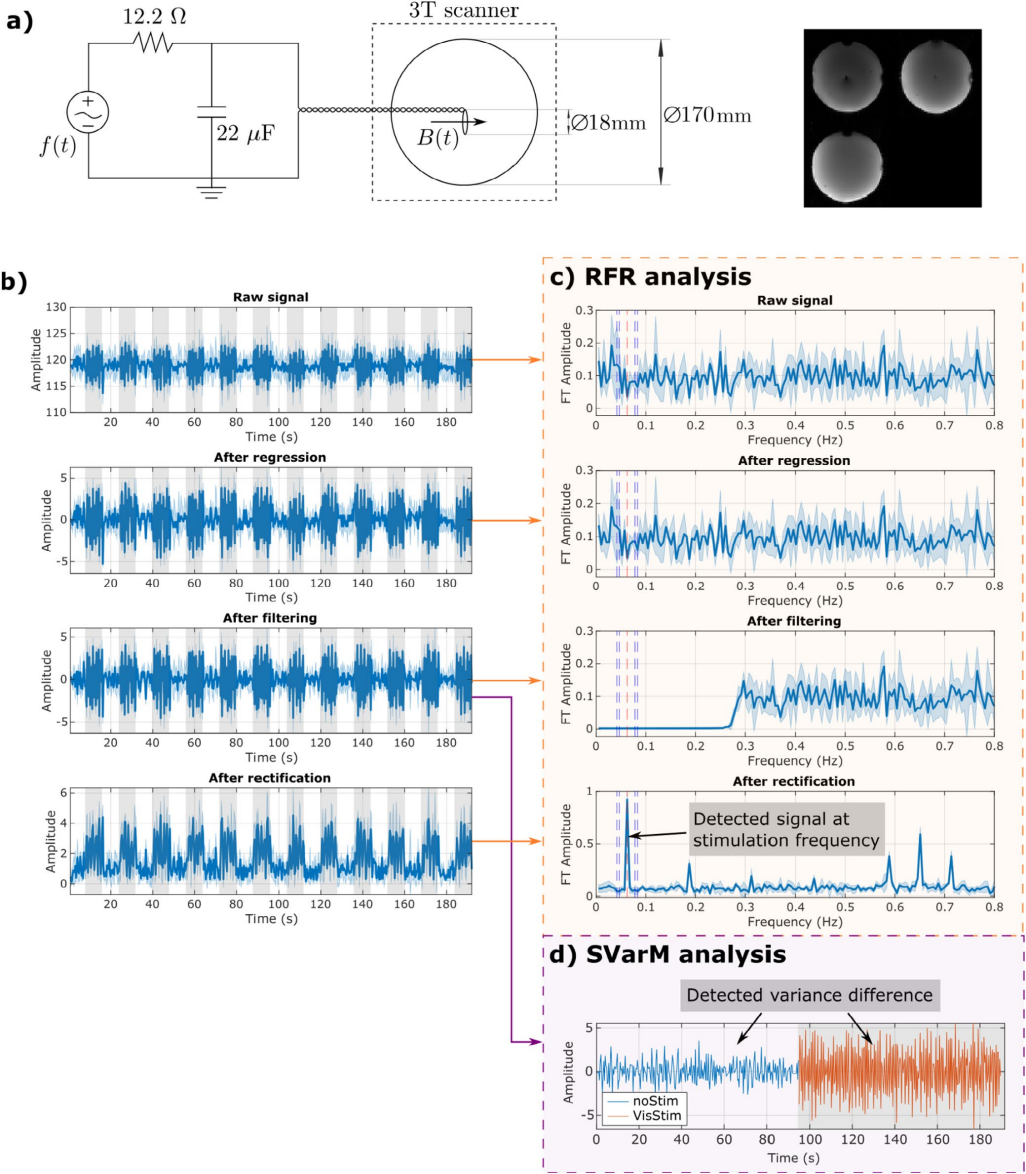
Postprocessing pipeline validation on phantom data. (a) Diagram of the electrical phantom setup. The field inside the loop *B*(*t*) is calculated through the Biot Savart Law. Bal‐SIRS image of the phantom is displayed on the right, (b) signal time course for the SL acquisition and output after regression, filtering, and rectification steps, (c) outputs of the RFR procedure in the frequency domain: the rectified signal highlights the detection at the block‐stimulation frequency of 0.0625 Hz, and (d) SVarM tests variance differences of the on and off stimulation periods.

To estimate the sensitivity limit for the Bal‐SIRS and Bal‐REX sequences, we used an electrical phantom previously described by Capiglioni et al. (2022). The phantom consisted of a 17‐cm acrylic sphere filled with a 0.07 mM MnCl_2_ saline solution. A loop coil inside the phantom is connected to a function generator and generates oscillatory magnetic fields oriented parallel to the main static field $B_{0}$. Figure 2a shows a schematic of the phantom setup.

The input waveform (Figure 2b) reproduced the expected neuronal magnetic field during the visual stimulation paradigm used in the human experiment. During each 8 s stimulation period, we applied a sinusoidal current described by $A\;\sin\left(\phi +\mathrm{\omega t}\right)$, where the initial phase ϕ was fixed at 0°, the angular frequency ω at 16 Hz, and A denotes the peak‐to‐peak voltage amplitude in mVpp. We performed two phantom experiments. First, to demonstrate the feasibility of detecting spin‐lock fMRI contrast and to validate the analysis pipeline, we applied a high‐amplitude input signal (*A* = 50 mVpp) to generate a strong oscillatory magnetic field. Second, to quantify the sensitivity limit, we systematically varied the input amplitude between 2 and 50 mVpp. The corresponding magnetic field amplitude, estimated using the Biot–Savart law for a current in a closed loop as previously proposed (Nagahara et al. 2013; Witzel et al. 2008), ranged from approximately 0.28–9 nT. The current flowing through the loop was measured both directly using an ammeter and indirectly via the voltage drop across a reference resistor using an oscilloscope. The two methods yielded consistent results. This field estimation approach was previously validated using the same phantom setup, where the calculated magnetic field amplitudes were found to be consistent with the observed SL‐fMRI contrast (Capiglioni et al. 2022). For both experiments, because the repetition time (TR) was not an integer multiple of the expected evoked neuronal response (1/16 s), successive measurements sampled a slightly different initial phase of the oscillation.

### 2.5 MRI Data Analysis

Image preprocessing for in vivo acquisitions included motion correction using MCFLIRT (FSL 6.04) (Jenkinson et al. 2002) and within‐subject coregistration with the high‐resolution image using SPM. For both the phantom and human data, the SL post‐processing combined elements of both the regression–filtering–rectification (RFR) method (Truong et al. 2019) and statistical variance mapping (SVarM) (Coletti et al. 2021), with adaptations to accommodate the present acquisition protocol. The RFR procedure first cleans the data from low‐frequency drifts and physiological nuances (mainly BOLD) and highlights the extra variance expected during stimulation. In the regression step, each voxel time course was modeled using the mean signal, a slow drift term, and for the in vivo experiments, a regressor corresponding to the hemodynamic response function at the stimulation frequency calculated as 1/16 s = 0.0625 s. A high‐pass filter was then applied to remove low‐frequency and residual hemodynamic components (Figure 2b). The filter cutoff was defined at 0.02 Hz, right above the controlled breathing frequency. The RFR procedure rectified voxel time courses, converting random‐phase signals into cyclic signals at the stimulation frequency. Contrast maps were computed as the power of the rectified signal at 0.0625 Hz (the block frequency). While RFR effectively isolates the periodic components associated with the task paradigm, it does not, by itself, provide voxel‐wise statistical inference. To address this, SVarM was subsequently applied to an intermediate RFR output (specifically, the regression‐filtered (RF) data) to perform statistical variance comparison with a Levene's test between stimulation and baseline blocks (Figure 2d).

In the phantom, this full analysis pipeline was applied to Bal‐Rex and Bal‐SIRS acquisitions measured as a function of the target field amplitude to determine the sensitivity limit. The sensitivity limit was defined as the lowest applied magnetic field amplitude for which the RFR contrast within the loop region remained significantly higher than the background signal (*p* < 0.01) in a same size ROI. In vivo, it was applied to five acquisitions: on‐resonance Bal‐REX (16 Hz), off‐resonance Bal‐REX (36 Hz), on‐resonance Bal‐SIRS (16 Hz), off‐resonance Bal‐SIRS (36 Hz), and conventional BOLD EPI.

To assess the robustness of the analysis with respect to parameter selection, we repeated the complete processing pipeline under multiple configurations. Specifically, we evaluated alternative high‐pass filter cutoffs (up to 0.3 Hz), applied image realignment using both SPM and FSL, and tested different temporal selections for the RFR analysis, including restricting stimulation and baseline blocks to their central 6 s, their initial 4 s, or the full block duration. We additionally varied regression parameters, including regressing or not the hemodynamic response function and modeling or omitting slow signal drifts. Unless otherwise specified, results are reported using the primary analysis pipeline described above. For transparency and reproducibility, all analysis scripts and statistical procedures are publicly available at https://github.com/milecap/MRIMEG_pipelines.

## 3 Results

### 3.1 Phantom Measurements: Validation of SL‐fMRI Detection Possibilities

To test the experimental feasibility of detecting a functional SL‐fMRI contrast tuned to our experimental paradigm, we started with a phantom experiment tuned to create an oscillating magnetic field at 16 Hz (activation). Figure 2c shows the result of each postprocessing step, clearly highlighting the peak at the block stimulation frequency after rectification and the higher variance when dividing in on and off blocks.

### 3.2 In Vivo Measurements of Neuronal Activity With MEG

**FIGURE 3:**
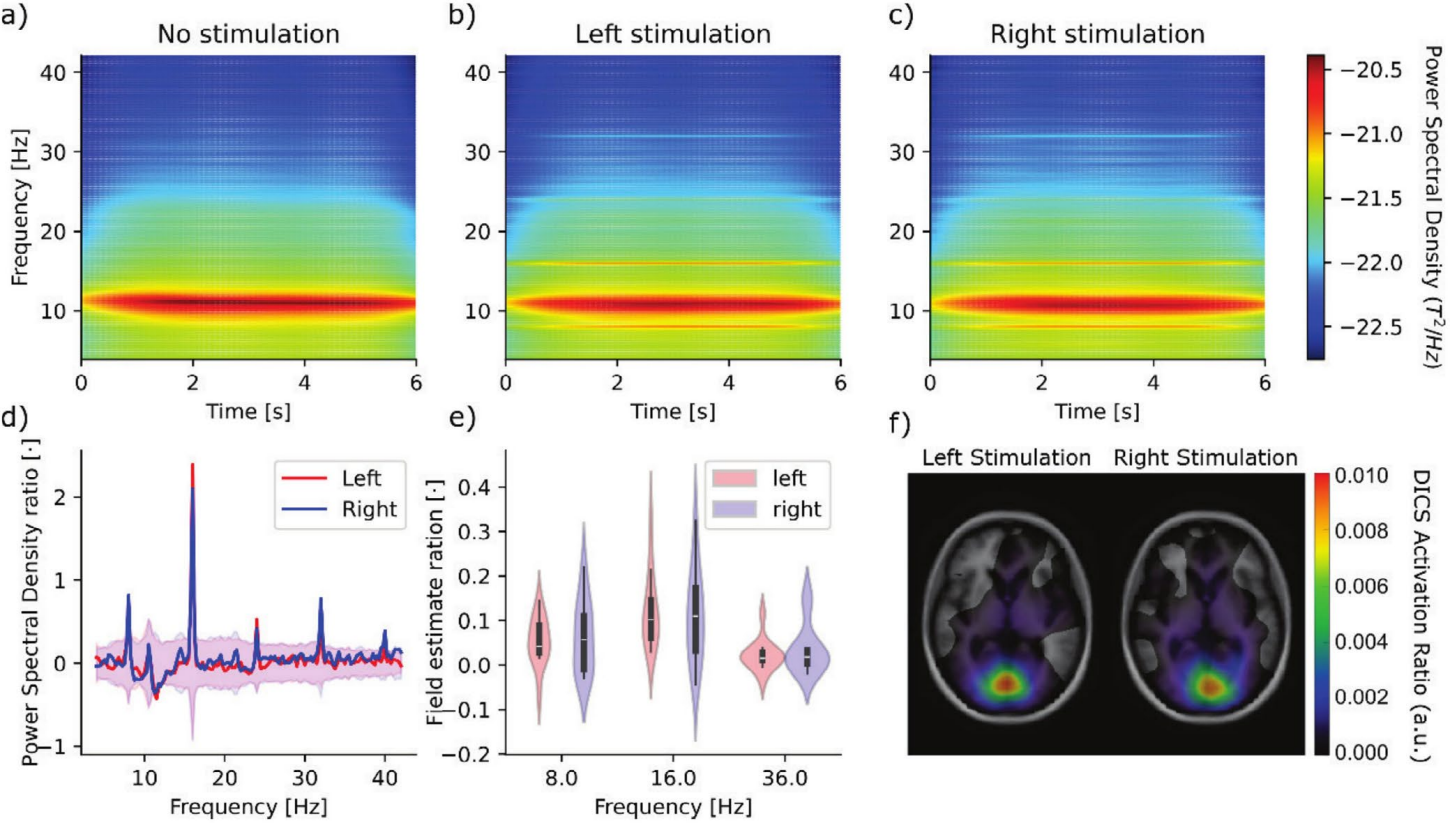
Group MEG visual stimulation results. Time–frequency maps of power spectral density from occipital sensors during (a) no flashing checkerboard stimulation, (b) right‐, (c) left‐field stimulation (Figure 1). Clear spectral peaks appear at 8, 16, and 24 Hz during stimulation, absent in the rest condition (logarithmic color scale), (d) ratio (stimulation vs. rest) of power spectral densities of occipital sensors, showing power peaks at 8, 16, 24, and 36 Hz (stimulation frequencies and harmonics), (e) violin plots of field ratios (stimulation—no stimulation, mean‐centered). Ratios are significant at 8 and 16 Hz, but not at 36 Hz (control), consistent with stimulus‐locked activity, and (f) statistically thresholded DICS activation maps averaged for all subjects for the ratio between stimulation and no stimulation in the left and right conditions.

MEG data was successfully analyzed for 12 out of 13 participants. The remaining participant was excluded due to incorrect acquisition timing. Figure 3a presents the results of the average frequency spectra across all subjects. MEG recordings confirmed robust stimulus‐locked activity in occipital sensors during visual flicker stimulation. The time–frequency maps (Figure 3a–c) showed clear spectral peaks at 8, 16, and 24 Hz during the 6 s epochs averaged across the stimulation periods for both stimulation sides, absent during rest. The power spectra ratio between stimulation/no‐stimulation periods, averaged across occipital sensors (Figure 3d), showed maximal entrainment at 16 Hz, consistent with the second harmonic of the flickering checkerboard, and a smaller peak at 8 Hz, but no response at the control frequency of 36 Hz (green arrow). Violin plots of field ratios (Figure 3e) confirmed that responses at 8 and 16 Hz were consistently above zero across participants, whereas no significant response was detected at the off‐resonance control frequency (36 Hz), validating this as an appropriate control frequency. In addition to the stimulus‐related peaks at 8, 16, and 24 Hz, a broader component around 10–12 Hz is visible, consistent with ongoing alpha‐band activity. Because this activity is not stimulus‐locked, it is not expected to contribute to the task‐related contrast identified by the RFR analysis.

### 3.3 In Vivo Measurements of Neural Currents With SL‐fMRI

**FIGURE 4:**
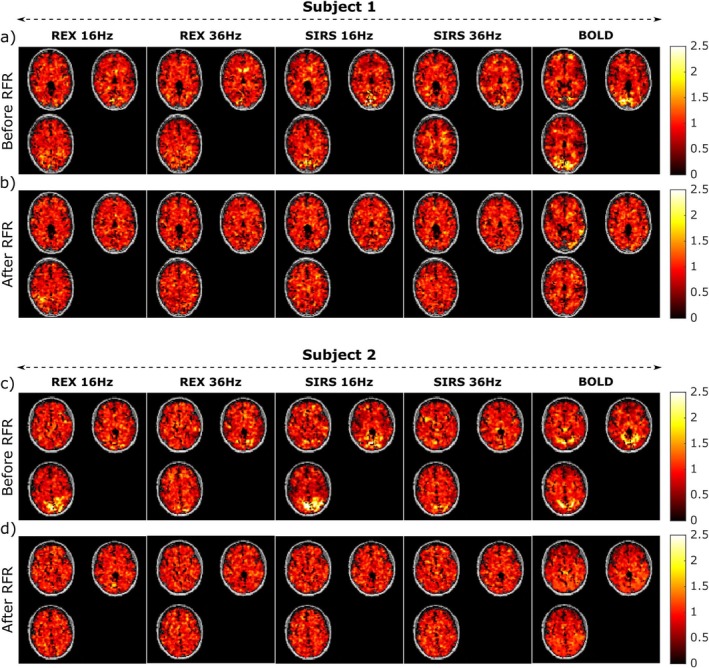
Regression–filtering–rectification effects on visual activation maps from spin‐lock fMRI. Unthresholded flashing checkerboard activation maps at the stimulation frequency (0.0625 Hz) are shown before (a, c) and after (b, d) the regression–filtering–rectification (RFR) procedure for on‐resonance Bal‐REX (16 Hz), off‐resonance Bal‐REX (36 Hz), on‐resonance Bal‐SIRS (16 Hz), off‐resonance Bal‐SIRS (36 Hz), and conventional BOLD EPI in two representative subjects. Maps were normalized in frequency and space aligned to a common scale for visualization.

Figure 4 illustrates representative normalized activation maps at the block‐stimulation frequency (0.0625) before and after RFR processing. Prior to RFR, all sequences show apparent activity in the visual cortex at the stimulation frequency, reflecting BOLD effects. Subject 1 (right‐field stimulation) and Subject 2 (left‐field stimulation) show correct lateralization of the pre‐RFR activity. The spatial extent and amplitude of the pre‐RFR activation vary across acquisitions, particularly in Subject 2 reflecting single‐subject and run‐to‐run variability. After RFR, most activations disappear, with some contrast remaining in the Bal‐REX on‐resonance (16 Hz) map of Subject 1, while no activation persists in the Bal‐SIRS, off‐resonance controls, or BOLD maps. In Subject 2, all activations are fully suppressed after RFR. This shows that the RFR procedure can correctly eliminate the hemodynamic effect. Nevertheless, contrary to the phantom experiments tuned at the stimulation frequency of 16 Hz, and despite the confirmation of this frequency activation from the MEG results, the in vivo results show no consistent SL‐fMRI induced contrast.

**FIGURE 5:**
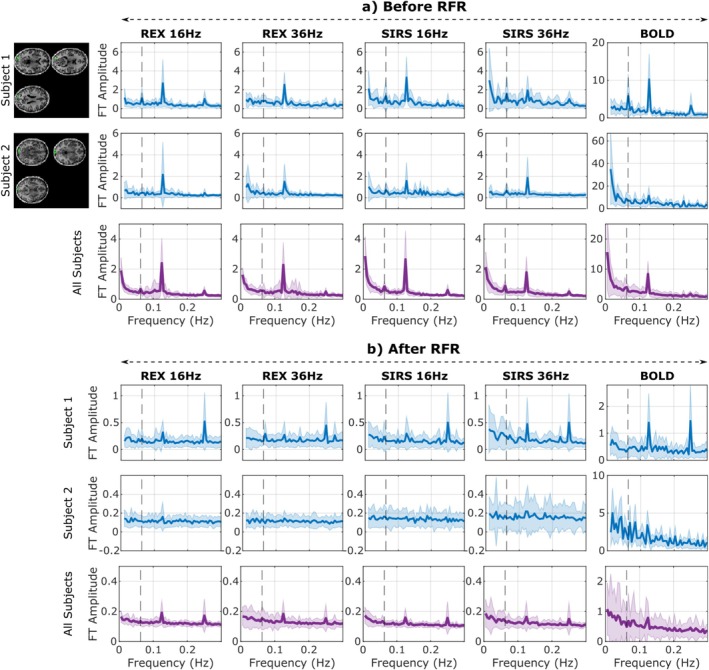
Spin‐lock fMRI power spectra in the visual cortex. Frequency‐domain data from two representative subjects and the group average within the anatomically defined visual cortex, shown (a) before and (b) after the regression–filtering–rectification (RFR) procedure. Results are presented for on‐resonance Bal‐REX (16 Hz), off‐resonance Bal‐REX (36 Hz), on‐resonance Bal‐SIRS (16 Hz), off‐resonance Bal‐SIRS (36 Hz), and conventional BOLD EPI acquisitions. The vertical dashed line at 0.0625 Hz marks the expected block‐stimulation frequency target.

Despite scattered highlighted areas after RFR (as for example Subject 1 Figure 4), neither Bal‐REX nor Bal‐SIRS showed consistent activation at 16 Hz relative to control frequencies. Power spectra within anatomically defined V1 ROIs (Figure 5) lacked peaks at the stimulation frequency following RFR processing, both at individual and group levels.

**FIGURE 6:**
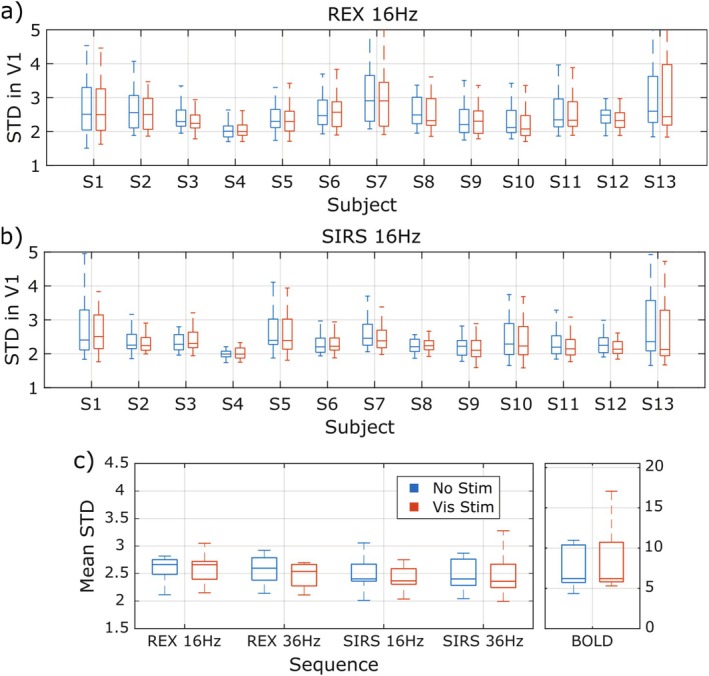
Group results of spin‐lock fMRI in visual cortex. Mean standard deviation for the left‐side‐stimulated and non‐stimulated periods within the visual cortex for (a) Bal‐REX, (b) Bal‐SIRS, and (c) averaged across all subjects and for each sequence: on‐resonance Bal‐REX (16 Hz), off‐resonance Bal‐REX (36 Hz), on‐resonance Bal‐SIRS (16 Hz), off‐resonance Bal‐SIRS (36 Hz), and conventional BOLD EPI acquisitions.

Because spin‐lock contrast is expressed in terms of signal variance, Figure 6 reports both the individual and group‐average distribution of signal variability across the 13 participants. Contrast was computed as the standard deviation of the functional signal within the primary visual cortex (V1), defined according to the Destrieux atlas for the individual subjects for (a) Ba‐REX, (b) Bal‐SIRS, and (c) averaged across subjects for side 1. No significant differences in variance were observed between stimulation and rest periods for either the spin‐lock or BOLD acquisitions. In addition, the standard deviation of the BOLD signal was consistently higher than that of the spin‐lock signal. No BOLD activation survived the applied preprocessing and statistical thresholds.

We observed no systematic differences across alternative analysis configurations or stimulation side (see Methods), and none of the tested parameter variations yielded consistent spin‐lock–specific activation.

### 3.4 Sensitivity Limits of Spin‐Lock fMRI to Detect Oscillating Currents

Having established basic feasibility with our first phantom tests (Figure 2) and verified the dominant neural oscillation frequency via MEG recordings (Figure 3), we were unable to reproduce consistent spin‐lock–based contrast in vivo (Figures 4, 5, 6). To assess whether this failure reflects physiological limits or methodological sensitivity constraints, we performed further phantom experiments to estimate the minimum detectable oscillatory current amplitude with spin‐lock fMRI and to compare this threshold to the average biomagnetic field strengths observed in MEG.

**FIGURE 7:**
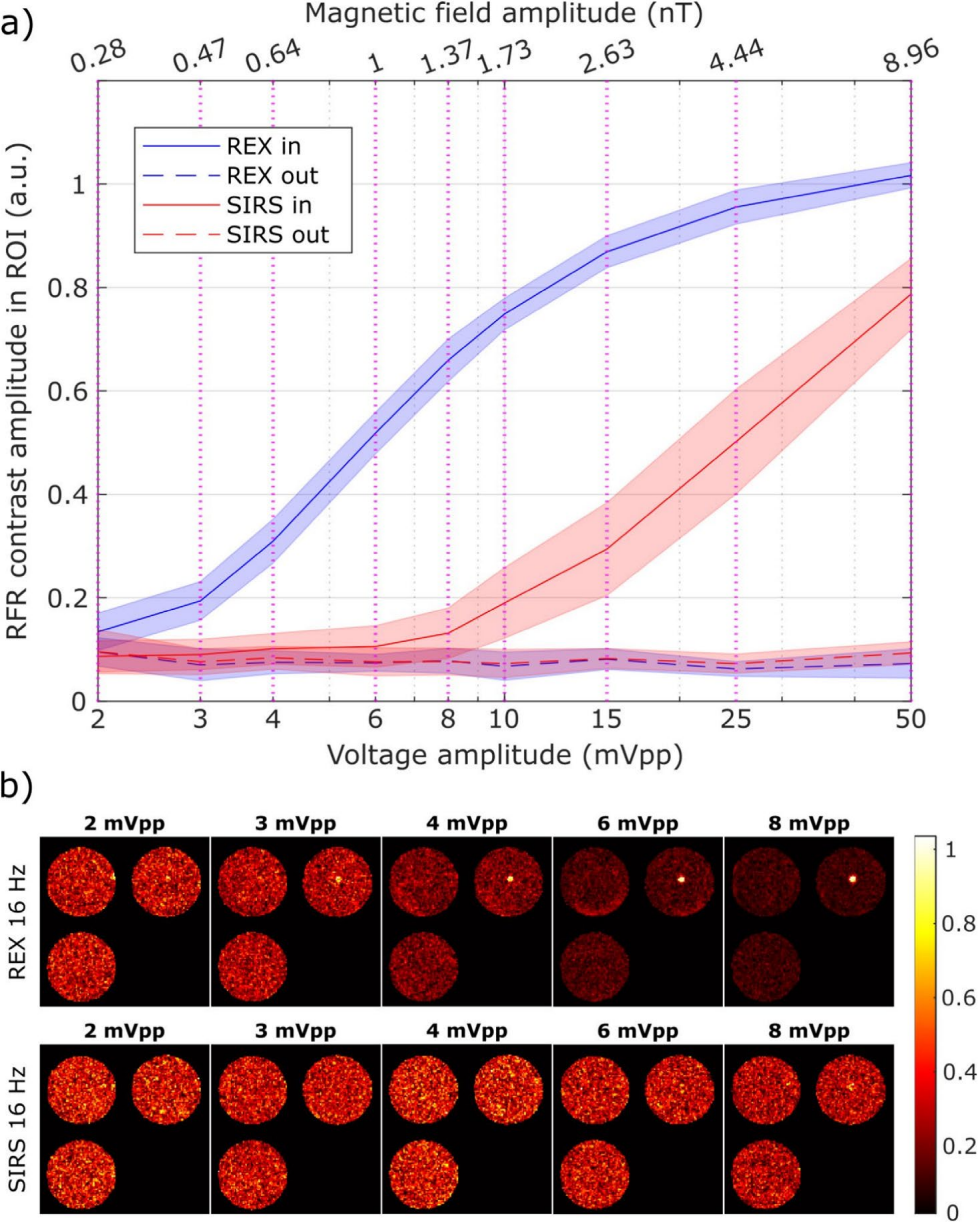
Phantom estimation of spin‐lock fMRI sensitivity limits. (a) RFR contrast as a function of the voltage amplitude for a ROI inside (in) and outside the phantom loop coil (out) for both SIRS and REX acquisitions. A *t*‐test comparing the contrast distribution between the in and out ROIs revealed significant differences (*p* < 0.01) in REX until 0.28 nT and SIRS until 0.6 nT. (b) RFR contrast maps for REX and SIRS signals as a function of the applied field's amplitude show both sequences' localization capabilities.

Figure 7 shows the resulting sensitivity analysis, where we systematically reduced the amplitude of a 16 Hz alternating current applied to the phantom coil and evaluated the resulting spin‐lock fMRI signal for both sequences. These experiments confirmed the higher functional sensitivity of the Bal‐REX acquisition relative to Bal‐SIRS, particularly as the imposed magnetic field approached the detection limit. We estimated sensitivity thresholds of approximately 0.2 nT for the Bal‐REX sequence and 0.6 nT for the Bal‐SIRS sequence. Results shown correspond to the RFR analysis pipeline, with comparable findings obtained using the SVarM pipeline.

We then compared these experimentally determined thresholds with magnetic field amplitudes estimated from the MEG recordings. Using the MEG‐based estimation procedure, we quantified the stimulus‐locked magnetic field amplitudes in the occipital cortex during visual stimulation. At the stimulation frequency of 16 Hz, left‐ and right‐field stimulation elicited an estimated group‐average local field amplitude of 0.073 ± 0.006 nT.

Together, these findings indicate that the measured detection limits of the spin‐lock acquisitions in our experimental configuration were ~3‐fold (REX) to 9‐fold (SIRS) higher than the average fluctuating field amplitude estimated from MEG. This sensitivity gap likely contributed to the lack of in vivo spin‐lock fMRI effects observed here.

## 4 Discussion

This study systematically evaluated the in vivo sensitivity of spin‐lock fMRI to detect neuronal magnetic fields in humans, using BOLD‐fMRI and MEG as benchmarks and calibrated phantom experiments on a 3T clinical MRI scanner. The main finding is that no significant SL‐fMRI activation was observed during a visual flashing‐checkerboard stimulation, despite robust biomagnetic and hemodynamic responses detected by MEG and BOLD. Quantitative comparison with phantom‐derived detection thresholds indicates that the neuronal magnetic field amplitudes estimated by MEG (~0.07 nT) lie below the minimal detectable levels of the implemented spin‐lock sequences (≥ 0.2 nT for balanced REX and ≥ 0.6 nT for balanced SIRS) under the present acquisition conditions.

The phantom experiments confirmed that both Bal‐REX and Bal‐SIRS sequences operated as expected (Bal‐REX with higher sensitivity than Bal‐SIRS) and that the analysis procedures were capable of detecting sub‐nanotesla oscillatory fields. The RFR and SVarM analysis pipelines to detect SL‐fMRI functional contrast provided consistent sensitivity estimates and effectively suppressed hemodynamic and low‐frequency noise in in vivo data, supporting the methodological validity of the negative finding. While balanced REX occasionally exhibited small residual fluctuations, neither sequence produced reproducible stimulus‐locked activation, preventing conclusions regarding relative in vivo performance differences between SIRS and REX.

The MEG experiments confirmed stimulus‐locked oscillatory responses at 8 and 16 Hz localized to the occipital cortex, consistent with previous visual entrainment studies. The estimated field strengths were approximately threefold lower than the minimal detectable fields in the phantom. Thus, the lack of SL‐fMRI activation is quantitatively consistent with the expected physical limits of the technique.

BOLD‐fMRI results revealed the expected contralateral visual activations at the expected frequency before the RFR procedure, providing further confirmation that the task design and subject compliance were appropriate and that cortical stimulation elicited genuine neuronal responses. The absence of spin‐lock effects in the same regions therefore reflects an intrinsic sensitivity limitation rather than lack of stimulation or analysis failure.

A limitation of this study is the use of echo planar imaging (EPI) readout instead of the previously used spiral acquisition (Truong et al. 2019). That study employed spiral readout and shorter (4 s) stimulation blocks, potentially shifting the dominant frequency content away from low‐frequency physiological confounds and improving signal‐to‐noise characteristics. Although SL contrast is encoded in signal variance rather than mean intensity, spiral acquisition may provide higher effective SNR and reduced hemodynamic sensitivity compared with EPI. Systematic cross‐validation of spiral and EPI readouts under identical conditions will be necessary to determine whether acquisition strategy alone can bridge the observed sensitivity gap. An additional potential drawback of the present sequence design is the use of a short repetition time (TR = 334 ms), which increases the number of repetitions within a fixed acquisition time but reduces longitudinal recovery and thus per‐volume signal amplitude. In the current design, we prioritized temporal sampling; however, the tradeoff between signal strength and statistical efficiency should be further evaluated in future studies.

Several additional factors may limit spin lock fMRI sensitivity in the present configuration. First, neuronal magnetic fields are vectorial and only the component parallel to *B*_0_ can contribute to spin‐lock interaction; a substantial fraction of the generated fields may therefore produce negligible SL effects. In addition, neuronal current sources generate dipolar magnetic field distributions that vary on sub‐voxel spatial scales. This may lead to partial cancelation of the effective magnetic field within a voxel and further reduce sensitivity. Second, the experiment was performed at 3T, where T₁ρ relaxation and *B*₁ inhomogeneities constrain achievable spin‐lock amplitudes. In particular, spatial variations in *B*₁ may shift the effective spin‐lock frequency away from its nominal value, reducing sensitivity to frequency‐specific neuronal signals. Although spin‐lock contrast exhibits a detection frequency bandwidth and is therefore not completely abolished by moderate frequency offsets (Capiglioni et al. 2022), such *B*₁‐related frequency mismatch may nevertheless contribute to the reduced sensitivity observed in vivo. In addition, *B*_0_ inhomogeneities introduce spatially varying off‐resonance effects that tilt the effective spin‐lock field away from its intended direction and, together with imperfect B₁‐induced tip‐down excitation, can generate residual signal oscillations. The balanced spin‐lock preparation used in this study is designed to compensate *B*_0_ and *B*_1_ imperfections; however, areas with strong field inhomogeneities can present residual signal fluctuations (Gram et al. 2022). Ultra‐low field implementations may offer improved sensitivity by diminishing *B*_0_ and *B*₁ effects. Third, the simple visual stimulation paradigm may have led to habituation or reduced attentional engagement, lowering response amplitude. Future studies using parametric modulation of stimulation frequency or intensity may help delineate detection thresholds more precisely. Finally, physiological and pathological neuronal events differ substantially in amplitude and synchrony. Epileptiform discharges, for example, arise from the hypersynchronous activity of large neuronal populations and can generate substantially larger electromagnetic signals than typical physiological oscillations (Cassarà et al. 2008; Charupanit et al. 2020; Rodionov et al. 2010). Although the present implementation does not achieve sufficient sensitivity to detect physiological oscillatory fields, pathological activity may represent a more favorable target for SL‐fMRI. Nevertheless, a quantitative comparison between epileptiform magnetic field amplitudes and the sensitivity thresholds measured in the present study remains unavailable, and further work will be required to establish whether such events fall within the detectable range of current spin‐lock methods.

Finally, it should be noted that the sensitivity thresholds reported here should not be interpreted as the fundamental sensitivity limits of Bal‐SIRS or Bal‐REX, but rather as the detection limits achieved with the specific phantom, in vivo configuration, and acquisition protocol used in this study. Future work will focus on improving sensitivity through alternative spin‐lock preparations and readout schemes, as well as systematically characterizing the magnetic field amplitudes and frequencies that can be detected under realistic in vivo conditions.

## 5 Conclusions

This study provides a multimodal evaluation of the detection limits of MR‐based neural current imaging mapping using spin‐lock fMRI. Despite the use of robust visual stimulation with careful control of acquisition and analysis procedures, SL‐fMRI did not detect stimulus‐locked neuronal activity at field strengths estimated by MEG, indicating that the current sensitivity threshold lies above the physiologically generated fields. These findings define a quantitative boundary for future methodological developments and contribute a reference for the continued search for direct MRI detection of neuronal currents.

## Funding

This work was supported by the International Society for Magnetic Resonance in Medicine, Research Exchange Grant 2022 and the Schweizerischer Nationalfonds zur Förderung der Wissenschaftlichen Forschung, CRSII5‐180365.

## Ethics Statement

The study was approved by the Ethics Committee of the University of Trento.

## Consent

All subjects provided written informed consent.

## Conflicts of Interest

The authors declare no conflicts of interest.

## Supporting information


**Figure S1:** Simulated dependence of spin‐lock signal modulation on spin‐lock duration (TSL). Standard deviation of the simulated signal as a function of TSL at 16 Hz for balanced SIRS (a) and balanced REX (b) and at 36 Hz for SIRS (c) and REX (d). Curves correspond to increasing amplitudes of the oscillatory target field (BNC = 0–0.05 nT). The dashed vertical line marks the selected TSL of 112 ms.

## Data Availability

The data presented in this study will be made available upon request to the corresponding author. The source code for data analysis will be made publicly available upon publication at: https://github.com/milecap/MRIMEG_pipelines.
